# Epidemiological Changes in Transthyretin Cardiac Amyloidosis: Evidence from In Vivo Data and Autoptic Series

**DOI:** 10.3390/jcm13175140

**Published:** 2024-08-29

**Authors:** Vincenzo Cianci, Alessio Cianci, Daniela Sapienza, Annalisa Cracò, Antonino Germanà, Antonio Ieni, Patrizia Gualniera, Alessio Asmundo, Cristina Mondello

**Affiliations:** 1 Department of Biomedical and Dental Sciences and Morphofunctional Imaging, Section of Legal Medicine, University of Messina, Via Consolare Valeria, 1, 98125 Messina, Italy; patrizia.gualniera@unime.it (P.G.); alessio.asmundo@unime.it (A.A.); mondelloc@unime.it (C.M.); 2 Department of Cardiovascular Medicine, Fondazione Policlinico Universitario A. Gemelli-IRCCS, Largo A. Gemelli 8, 00168 Rome, Italy; alessiocianci.1998@gmail.com; 3 Department of Biomedical Sciences and Morphological and Functional Imaging, Diagnostic and Interventional Radiology Unit, University Hospital Messina, 98168 Messina, Italy; annalisacraco@hotmail.it; 4 Zebrafish Neuromorphology Lab, Department of Veterinary Sciences, Via Palatucci snc, University of Messina, 98168 Messina, Italy; agermana@unime.it; 5 Department of Human Pathology in Adult and Developmental Age “Gaetano Barresi”, Section of Pathology, University of Messina, 98125 Messina, Italy; antonio.ieni@unime.it

**Keywords:** cardiac amyloidosis, transthyretin, cardiac amyloidosis epidemiology, cardiomyopathy, ATTR amyloidosis, amyloid fibrils, apical sparing, ATTR amyloidosis postmortem diagnosis

## Abstract

Cardiac amyloidosis is an infiltrative disease that causes progressive myocardial impairment secondary to amyloid fibril deposition in the extracellular space of the myocardium. Many amyloid precursors, including transthyretin protein, are known to determine cardiac damage by aggregating and precipitating in cardiac tissue. Transthyretin cardiac amyloidosis may be either caused by rare genetic mutations of the transthyretin gene in the hereditary variant, or may arise as a consequence of age-related mechanisms in the acquired form. Although it has been labeled as a rare disease, in recent years, transthyretin cardiac amyloidosis has stood out as an emerging cause of aortic stenosis, unexplained left ventricular hypertrophy and heart failure with preserved ejection fraction, particularly in the elderly. Indeed, the integration of data deriving from both in vivo imaging techniques (whose advancement in the last years has allowed to achieve an easier and more accessible non-invasive diagnosis) and forensic studies (showing a prevalence of amyloid deposition in cardiac tissue of elderly patients up to 29%) suggests that cardiac amyloidosis is a more common disease than traditionally considered. Thanks to all the improvements in non-invasive diagnostic techniques, along with the development of efficacious therapies offering improvements in survival rates, transthyretin cardiac amyloidosis has been transformed from an incurable and infrequent condition to a relatively more diffuse and treatable disease, which physicians should take into consideration in the differential diagnostic processes in daily clinical practice.

## 1. Introduction

Amyloidosis is a systemic disorder arising from the deposition of misfolded proteins in the extracellular space that can theoretically affect any organ, including the heart [1]. The accumulation of the insoluble proteinaceous material, named amyloid, in the extracellular space of myocardial tissue determines a structural damage, which gives rise to a cardiomyopathy that presents with a hypertrophic phenotype with increased biventricular wall thickness and stiffness, and evolves towards a restrictive phenotype in the advanced phases of the disease [2]. Amyloid deposits lead to an increase in extracellular volume and consequent disruption of myocardial architecture, thus causing a progressive functional impairment characterized by diastolic and systolic disfunction, with heterogeneous clinical manifestations [1].

In vivo, more than 35 proteins have been identified as amyloidogenic precursors, but just 9 of them may produce significant cardiac disease, depositing in the interstitial space between cardiac myocytes. Among them, diagnosed cardiac amyloidosis (CA) usually derives from the accumulation of amyloid fibrils made up of monoclonal immunoglobulin light chain (AL-CM) or transthyretin proteins (ATTR-CM) [3].

Indeed, ATTR amyloidosis results from the progressive accumulation of misfolded transthyretin proteins that aggregate into insoluble amyloid fibrils; it derives either from altered biological processes, not yet completely characterized, linked to aging in its acquired variant (wild-type ATTR, ATTRwt), or from specific genetic mutations in its hereditary form (variant ATTR, ATTRv) [4].

With the present narrative review, the authors aim to explore the epidemiological changes of ATTR-CM, which has evolved in the last years from a rare condition to a quite frequent disease, gathering evidence from original research, reviews and metanalyses provided on PubMed and Scopus, without temporal limitation.

## 2. Pathophysiology

Human transthyretin (TTR), also known as prealbumin, is a protein synthesized primarily in the liver, choroid plexus and retinal pigmented epithelium. Although hepatocytes represent the main site of production of circulating TTR, this protein has also been isolated in the pineal gland, neurons, renal cells and pancreatic island cells, and its expression is precisely regulated by nuclear or mitochondrial transcription factors and steroid hormones [5]. 

Human TTR is a homo-tetrameric protein that derives from the assembly of four monomers of TTR in a complex tridimensional structure with a molecular weight of 55 kDa, characterized by a central hydrophobic core that constitutes the ligand binding site [6]. Indeed, TTR is mainly responsible for transporting thyroxine and retinol (vitamin A) complexed with retinol-binding protein, through circulating blood and cerebrospinal fluid. In addition, the hydrophobic channel of TTR may also accommodate other substances, such as high-density lipoproteins through apolipoprotein A1, lutein and other carotenoids or norepinephrine oxidation products [7]. Beyond its transporting function, TTR contributes to neuroprotection in various pathological conditions, such as ischemia and Alzheimer’s disease, and has a role in memory stabilization, regulating hippocampal processes and insulin secretion [8,9]. 

The physiological functions of TTR rely on the stability of its tridimensional shape, which depends mostly on intra- and intermolecular hydrogen bonds, and on the redox state of the environment, with a proper balance between reducing and oxidative agents [10]. TTR’s structure may be altered by destabilizing gene mutations, which represent the main pathogenetic cause of ATTRv [11]. However, TTR misfolding may occur with senescence in absence of gene mutation, through mechanisms not yet fully understood. In the case of ATTRwt, the imbalance of redox state towards oxidation may play a key role in this pathogenetic process, affecting the spatial conformation of N-terminus aminoacidic residues and thus determining tetramer instability [10,11,12]. 

Structural impairment leads to the homo-tetramers’ progressive decomposition to dimers and consequently to unfolded monomers, which are then able to initiate the process of amyloidogenesis. Monomers aggregate into heterogeneous and dynamic oligomers, which finally assemble into insoluble fibrillary material with an aberrant quaternary structure, characterized by an ordered cross β-sheet conformation resistant to proteolytic enzyme activity [13]. The accumulation of proteinaceous material in the interstitial space determines the alteration of the extracellular matrix architecture, leading to an imbalance in homeostatic processes and inducing a pro-inflammatory state, which finally results in tissue disruption and functional damage [10]. 

## 3. Anatomopathological Findings 

### 3.1. Gross Findings

Amyloid deposition may theoretically affect any portion of the heart, from atrial and ventricular walls to the valves and the vessels [14]. 

Atrial infiltration is rather frequent in cardiac amyloidosis and presents with a patchy involvement that causes morphological alterations, contributing to the characteristic severe dilation observed in the disease [15].

Ventricular infiltration determines an increase in thickness and stiffness of myocardial walls, which assume a waxy appearance at gross examination. Although in the early phase of the disease, amyloid accumulation may predominantly affect the interventricular septum, mimicking the behavior of hypertrophic cardiomyopathy, the whole myocardium is involved in the late stages of the pathology, evolving towards symmetric concentric hypertrophy [16,17]. Nevertheless, the presence of normal left ventricular wall thickness (LVWT), which is defined in cardiac amyloidosis as LVWT < 12 mm, does not necessarily exclude amyloid cardiac infiltration and does not differ in terms of prognostic outcomes when compared with the hypertrophic phenotype [18,19]. Indeed, on a forensic perspective, it is fundamental to shed light on ATTR-CM as an increasing cause of sudden cardiac death (SCD) in elderly patients, as myocardial infiltration may lead to electrical instability independently of the presence of LV hypertrophy, in relation to patchy myocardial accumulation and circumscribed microscopical fibrosis [20,21].

Amyloid deposition may also occur in the endocardial layer of the heart, including the valvular coating, which may assume, as a consequence, a granular and sandpaper-like appearance. In addition, epicardial involvement may be present, manifesting as diffuse whitish nodules on the heart’s surface [22]. 

Importantly, amyloid accumulation may involve epicardial coronary arteries layers, which, nevertheless, have not been directly connected to the obstruction of epicardial vessels and thus to myocardial ischemia [23]. However, obstruction may occur in the vasa vasorum of the epicardial coronary arteries [24]. The same tendency has been observed in intramural coronary microcirculation, where amyloid deposition may lead to obstructive depositions and may be responsible for coronary microvascular disfunction and anginal symptoms in patients with CA [25]. 

### 3.2. Histological Findings

Amyloid is characterized by a β-sheet conformation, which is responsible for its peculiar histological properties. Indeed, it binds linear dyes such as Congo red and shows the pathognomonic apple-green birefringence when examined under cross-polarized light [26]. Nevertheless, other stains may be used to detect amyloid accumulation in cardiac tissue, such as sulfated alcian blue, which highlights the mucopolysaccharides connected to amyloid, or fluorochrome dyes, which determine fluorescence in dark field microscopy [27,28]. 

Amyloid deposition involves predominantly the interstitial space of the subendocardial and mid layers of the myocardial wall and usually exhibits a patchy pattern [29]. Extracellular accumulation may occur either with a peri-cellular pattern, if it surrounds the single myocytes, or with a nodular pattern [30]. Moreover, different histopathological features of amyloid fibrils between ATTRv and ATTRwt have been described. Regarding morphological characteristics, Bergstrom et al. found that a subject with ATTRwt showed short and rigid fibrils in opposition to long straight parallel fibrils seen in patients with ATTRv. These findings may be related to the presence of underlying different pathogenetic mechanisms, which may generate different amyloid fibrils [29]. 

Once amyloid deposition has been identified, the characterization of amyloidogenic protein is a crucial next step, as it allows to initiate a tailored treatment in all cases where non-invasive diagnosis alone is inconclusive [31]. The identification of the amyloid fibril type relies on immunohistochemical staining with a panel of specific antibodies, using immunoperoxidase or immunofluorescence techniques [32].

However, considering that immunohistochemistry may not give univocal results in about three out of ten of cases, either the analysis by mass spectrometry of mass and charge of peptide segments compared with a panel of sequenced proteins or the use of immunogold electron microscopy, may be valid further alternatives, showing the highest specificity and sensitivity for amyloid characterization [33,34]. 

## 4. Epidemiological Changes

ATTR-CM has been historically deemed a rare disease, affecting fewer than 0.5 people in 100,000. Indeed, in addition to an inadequate knowledge of its pathophysiological mechanisms, an invasive diagnosis through endomyocardial biopsy was required, which limited its recognition over the past years [35]. 

In the past years, the most common form of cardiac amyloidosis was represented by AL-CM, an acquired disease characterized by the overproduction of monoclonal components by the plasma cellular elements of the bone marrow, determining in most cases a systemic rapidly progressive disease, with symptomatic cardiac involvement being a sign of poor prognosis. Nevertheless, in the last decade, registries of patients diagnosed with cardiac amyloidosis displayed a progressive and incessant increase in ATTR-CM diagnosis, to the extent that, nowadays, it represents the most prevalent form of CA [36,37].

The reason for this trend reversal lies in the development of a non-invasive diagnostic algorithm of ATTR-CM, based on disease detection through the application of scintigraphy with a bone tracer and cardiac magnetic resonance (CMR), which has reduced the need for resorting to anatomopathological confirmation to demonstrate the presence of amyloid fibrils in about 70% of cases [38]. Thus, a more accessible diagnosis, associated with a greater awareness of the disease from the physicians fostered by the development of tailored disease-modifying therapies, has been responsible for the observed considerable increase in ATTR-CM prevalence rates [39]. 

### 4.1. Main Population Features

#### 4.1.1. Sex Distribution

ATTR-CM, both in the ATTRwt and ATTRv form, has been widely described as a condition predominant in male subjects, with an in vivo prevalence rate of 80% and incidence increasing with aging, especially for ATTRwt [40]. 

Specifically, evidence from the Transthyretin Amyloidosis Outcomes Survey (THAOS), a global observational survey studying various aspect of transthyretin amyloidosis, described a clear prevalence of ATTRwt in male patients (94% of 1386 affected subjects), with a mean younger age at diagnosis compared with female subjects [41]. Similarly, a sex-based clinical analysis of 2790 patients with ATTRv, included in the THAOS registry, highlighted male prevalence (7 out of 10 patients), with a younger age at diagnosis and a greater extent of myocardial involvement [42].

Moreover, considering only symptomatic patients, the majority were represented by men (70.8%), with a mean age at symptoms onset of 56.6 years; in contrast, considering only asymptomatic gene carriers of TTR mutation, 58.5% were female, with a mean age of 41.9 years [43].

The reasons for this significant difference are not yet completely understood. Both genetic or biological factors, including a potential protective role of female sex hormones until menopause, and environmental triggers may play a key role [44]. Moreover, the lack of sex-tailored diagnostic cut-offs for left ventricular hypertrophy may represent a further selecting bias during the diagnostic process, underestimating the prevalence rates in women [3].

In this regard, autoptic case series in elderly unselected patients (aged ≥ 80 years) demonstrated that amyloid myocardial detection rates do not exhibit remarkable sex differences, with only a minor percentage of patients manifesting with heart failure symptoms or sudden cardiac death [45,46]. However, amyloid infiltration is more severe in men than in woman (10% of affected males had extensive cardiac amyloid deposition, which was present only in 1% of females), which coincided with a major clinical severity of the disease in males [47].

#### 4.1.2. Geographic and Ethnic Distribution

THAOS represents the biggest ongoing international and observational registry. It was established in 2007, and planned to describe the genotype/phenotype features, the natural history and the impact of disease-modifying therapies in patients with TTR amyloidosis, both ATTRwt and ATTRv, including both symptomatic subjects and asymptomatic gene mutation carriers [43]. 

The latest analysis of the registry, covering a 15 year period from 2007 to 2022, carried out by Gentile et al. [43], included more than 6000 patients worldwide and showed that Val30Met represents the most diffuse genotype in South America (78.6%), Japan (74.2%) and Europe (62.2%), being responsible in the majority of cases for a predominantly neurological or a mixed phenotype. Specifically, Japan, Brazil and northern Portugal are considered endemic areas for Val30Met “early-onset” (third/fourth decade of life), manifesting with a predominantly neurologic phenotype and with cardiac disease generally circumscribed to electrical conduction system alterations [43]. 

Moreover, according to the THAOS database, the second most frequent mutation in Europe is Ile68Leu, which generates a predominantly cardiac phenotype, with most subjects affected in Italy, where this mutation represents the most diffuse genetic variant (16.1% of the patients with ATTRv) [48]. Regarding Italy, another study from Rapezzi et al. stated that, among an overall ATTRv population of 186 Caucasian patients with Italian descent living in non-endemic areas, Val30Met with a “late-onset” phenotype was the most common mutation (24.8%), while Ile68Leu resulted as the most diffuse variant in patients with an exclusively cardiac phenotype and, globally, the second most frequent mutation [49].

ATTRwt is the most prevalent form in North America (56.2%), while the most represented TTR gene mutation in the United States is Val122Ile (p.Val142Ile), particularly in African-American ethnicity [47,50]. Beyond the THAOS database, Lahuerta Pueyo et al. estimated that the mutation Val122Ile, seen in people of African descent, may be the most common mutation of the TTR gene, with global prevalence among the general population of 0.3% [51,52].

### 4.2. Evidence of Epidemiologic Evolution

#### 4.2.1. Registries of Diagnosed Patients

Current knowledge about the epidemiology of ATTR-CM is based on studies and registries of diagnosed patients, which show a paradigmatic epidemiological evolution over the last two decades [37].

Lauppe et al. [53] carried out a prevalence study combining multiple national health registers in Sweden, finding out that the prevalence rates of ATTR-CM rose progressively from 1.0 per 100,000 to 5.0 per 100,000 person-years, from 2008 to 2018. The same tendency was observed in other Nordic countries, especially in Norway, with a steady increase in the prevalence rate up to 3.7 per 100,000 person-years during the same period of time [54]. Data from the regional population-based registry in Tuscany updated to 2022 showed a prevalence of 90.3 per 1,000,000 persons for ATTRwt and 9.5 per 1,000,000 persons for ATTRv [55]. 

Gilstrap et al. [56] investigated how the incidence and prevalence of cardiac amyloidosis has evolved between 2000 and 2012 in patients hospitalized for heart failure among the Medicare beneficiaries in the United States. They concluded that there has been a relevant rise in the prevalence rate (8 to 17 per 100,000 person-years) and incidence rate (18 to 55 per 100,000 person-years) from 2000 to 2012, particularly in specific subgroups of patients, such as elderly men. 

The rise in the median age at diagnosis and the higher incidence in males suggests the predominant role of ATTRwt amyloidosis in determining this increase [57,58]. Further confirmation of this trend derives from anatomopathological studies conducted in Japan by Naiki et al. [58]. Analyzing with immunohistochemistry techniques 4119 biopsy samples received from April 2018 to July 2022, the incidence of ATTR-CM was 54.9% of the total. In particular, taking only cardiac biopsies into account, out of 2208 cases, 1503 were ATTR positive, with a steep increase in the number of transthyretin-positive cardiac biopsy cases in the last year of the study compared with the first one. 

The main data are summarized in Table 1.

#### 4.2.2. Epidemiological Data from Specific Clinical Settings

Epidemiological studies have also been performed in specific clinical settings that have been associated with cardiac amyloidosis, such as heart failure with preserved ejection fraction (HFpEF), severe aortic stenosis in old patients, unexplained or disproportionate left ventricular thickening and surgery for bilateral carpal tunnel syndrome. 

Aimo et al. [59] performed a meta-analysis of several screening studies and demonstrated that screening programs for cardiac amyloidosis in selected clinical settings led to the identification of a relatively high number of cases, enforcing the concept that cardiac amyloidosis is a more frequent disease than traditionally deemed. They described an overall prevalence of cardiac amyloidosis of 12% in patients with HFpEF [60,61,62,63,64], and a prevalence of 7% in patients with bilateral carpal tunnel syndrome, which increased to 14% in elderly individuals without occupational risk factors and with coexisting increased left ventricular thickness [65,66,67]. 

Similar data emerged from the analysis of several screening studies performed in patients with severe aortic stenosis who were candidates either for surgical or trans-catheter valve replacement (TAVR), showing a mean prevalence of 8%, above all regarding elderly males undergoing TAVR [68,69,70]. 

Moreover, screening exams for cardiac amyloidosis were performed in patients with previously diagnosed hypertrophic cardiomyopathy, demonstrating a prevalence of misdiagnosis of cardiac amyloidosis in 7% of the subjects [71].

#### 4.2.3. Epidemiological Data from Autoptic Studies

Further emblematic data derive from postmortem examinations of unselected elderly individuals [72,73,74,75]. 

Lie et al. [72] performed autoptic exams on the hearts of 237 subjects (93 men and 144 women) aged between 90 and 105 years. Amyloid deposits were detected in one-fifth of patients in at least 25% of myocardial samples; however, in this study, no data were provided about the clinical impact and the potential correlation between amyloid accumulation and the cause of death. 

Roberts et al. [73] carried out a study about the pathological cardiac changes of 490 unselected elderly individuals aged more than 80 years old. About half of the subjects suffered from cardiac death. Among them, cardiac amyloidosis was identified macroscopically and histologically in ventricular and atrial myocardium and it was considered responsible for the decease of the patients in 10% of cases. Moreover, despite not having macroscopic evidence of amyloid deposition, histologic examination revealed the presence of minute agglomerated amyloid fibrils, not determining any manifest cardiac symptom, in a great number of other patients in the cohort. 

Furthermore, Tanskanen et al. [74] conducted autoptic examinations on 256 elderly patients, searching for the presence of amyloid deposition on Congo red-stained myocardial specimens. Amyloid fibrils were identified in 63 subjects (25% of the study population), with an increase in incidence rate in relation to higher age at death, and all the histologic specimens showed a positive reaction to the anti-TTR immunohistochemistry characterization. Cardiac amyloidosis was defined severe because of the great quantity of amyloid fibrils found in the examined samples in 11% of these individuals, and thereby likely responsible for the clinical outcome.

Porcari et al. [75] evaluated the prevalence of cardiac amyloidosis in a study population of 56 unselected patients who died at a median age of 86 years undergoing autoptic study, correlating the anatomopathological findings to their clinical history. Cardiac amyloidosis was diagnosed in 24 patients, corresponding to 43% of the individuals, and was deemed the principal determinant of death in 8 patients (14% of the study cohort), with an increase in the prevalence rate with aging (only 25% of the individuals between 75 and 79 years old). Amyloid infiltration especially concerned the atria (96%), the left ventricle (88%), the interventricular septum (80%) and the right ventricle (71%). In this regard, right ventricular involvement was associated with a higher amyloid burden. 

The amyloid deposits were further analyzed through immunohistochemical techniques with kappa and lambda light chain antibodies and anti-TTR antibodies, revealing a similar incidence rate between AL-CA and ATTR-CA. As the histological results were matched with the clinical data of the patients, an association emerged between cardiac amyloidosis and the presence of clinical or echocardiographic “red flags”, such as heart failure with preserved ejection fraction, atrial fibrillation, biventricular hypertrophy and typical electrocardiographic features as low QRS complex voltages or incongruity between left ventricular mass index at echocardiogram and QRS complex voltage [75]. 

In conclusion, a pooled assessment of these postmortem studies showed an overall prevalence of 21% in unselected individuals aged more than 75 years old [59]. 

Moreover, Corwell et al. [76] performed autopsies on 85 patients aged 80 years or older to detect the presence of transthyretin deposition in cardiac specimens, with positive findings in 25% of the subjects, mostly of small extent and with concomitant extracardiac involvement. Comparing this information with clinical data available, a higher frequency of atrial fibrillation and heart failure was reported in these patients, even if it did not reach statistical significance.

In this regard, however, further autoptic examinations were performed to investigate the potential relation existing between cardiac amyloidosis and specific clinical settings. Mohammed et al. [77] showed a higher prevalence of cardiac wild-type transthyretin deposition in left ventricular autopsy samples from patients with antemortem diagnosis of HFpEF, rather than in cardiac specimens from healthy controls matched for age of death and sex. Amyloid accumulation was deemed the primary cause of HFpEF in 5% of patients, above all men, when reaching a moderate or severe level of interstitial deposition. 

The main data are summarized in Table 2.

## 5. Cardiac Clinical Manifestations 

ATTR-CM arises with prevalent cardiac involvement in its senile form (ATTRwt). On the contrary, ATTRv may exhibit a more complex clinical picture, characterized by both polyneuropathy and cardiomyopathy, with different levels of gravity of peripheral nervous system or cardiac involvement depending on the type of mutation and the degree of penetrance [78]. In particular, four TTR gene mutations (Val122Ile, Thr60Ala, Leu111Met and Ile68Leu) are known to exhibit a predominantly cardiac phenotype [49].

Pan-cardiac amyloid deposition determines heterogeneous manifestations, ranging from HFpEF, which embodies the classic disease presentation, up to severe aortic stenosis and arrhythmias. Thus, differential diagnosis with similar conditions is not always a simple process [79,80]. 

Exertional dyspnea and lower limb edema are common symptoms in the setting of HFpEF, caused by diastolic impairment with a ventricular restrictive filling pattern. In addition, sporadic episodes of chest pain, mimicking acute coronary syndromes [81], may occur as a result of microvascular disfunction or aortic stenosis. 

Amyloid accumulation in the electric conduction system may produce atrioventricular (AV) blocks, leading to dizziness or syncopal events [3]. 

Tachycardia is a common symptom that may represent a compensation mechanism to maintain an adequate cardiac output, which tends to be reduced in ATTR-CM due to the ventricular stiffness with a fixed low end-diastolic volume. Moreover, palpitations may derive from the development of atrial fibrillation, a quite frequent comorbidity deemed a consequence of amyloid infiltration of the atria, along with the presence of elevated ventricular filling pressure and subsequent bi-atrial enlargement [4,78]. 

Ventricular tachyarrhythmias may occur as a consequence of amyloid infiltration of the electrical conduction system, microvascular ischemia or fibrotic alterations, leading to the formation of intraventricular re-entrant circuits regardless of the presence of LV hypertrophy [80,82]. 

Electrical instability determined by AV blocks and ventricular tachycardias could lead to electromechanical dissociation, representing the principal pathophysiological foundation of SCD [83]. 

## 6. Red Flags

In consideration of the variegated cardiological clinical picture of ATTR-CM at presentation, relevant clues for the diagnosis derive from the association of peculiar extracardiac clinical manifestation and first-line diagnostic tools, such as serum blood markers, electrocardiogram (ECG) and echocardiography [3]. The identification of these findings, known as “red flags” as they demonstrate a strong epidemiological link with cardiac amyloidosis, may be useful for physicians to raise the suspicion of amyloidotic cardiomyopathy in order to undertake the specific diagnostic pathway for the detection of the disease [4,84]. In particular, a left ventricular wall thickness ≥ 12 mm, associated with at least one cardiac or extracardiac red flag, should prompt the physician to start the diagnostic process [3].

### 6.1. Extracardiac Manifestations

In terms of extracardiac involvement, one of the most typical findings is represented by bilateral carpal tunnel syndrome, which arises in about 15% of patients with ATTRwt and may precede cardiomyopathy up to ten years [85]. Other pathological conditions potentially associated with amyloid accumulation are bicep tendon rupture, often asymptomatic and clinically evident as the so-called “Popeye sign”, and lumbar spinal stenosis, causing back pain and a sensory-motor syndrome that affects the lower limbs, as a consequence of nervous compression at the lumbar level. [4]

Further red flags for ATTR-CM are linked to the development of autonomic nervous system dysfunction, arising with orthostatic hypotension, decline of systolic blood pressure with tendency to normo- or hypotension in patients with previously diagnosed arterial hypertensive disease, or gastrointestinal disorders, such as constipation or diarrhea [86]. 

Peripheral nervous system involvement with progressively evolving sensory-motor polyneuropathy is distinctive of ATTRv [87]. An analysis from the THAOS registry concerning more than 6000 patients [43] showed a prevalence of 24.5% of mixed (cardiac and neurologic) phenotypes, with mainly the cardiac phenotype present in 31.9% of cases and predominantly neurologic in 38.7% of subjects. 

### 6.2. First-Line Diagnostic Tools

#### 6.2.1. Electrocardiography

**Figure 1:**
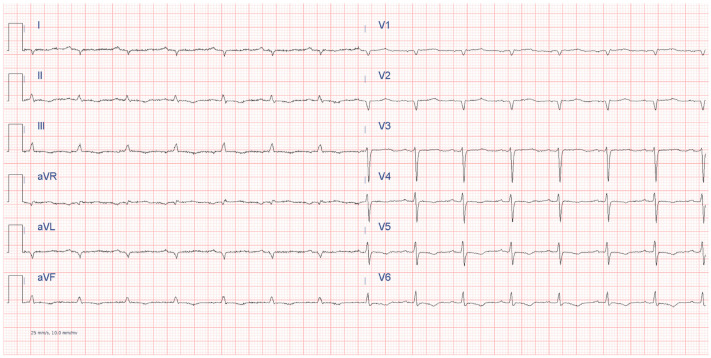
Electrocardiogram showing typical features of cardiac amyloidosis. Low peripheral QRS complex voltages and pseudo-infarct pattern in V1–V2 with poor R wave progression in the precordial leads and diffuse ventricular repolarization abnormalities (inverted T waves in inferior leads and in V4–V6).

One of the first steps in approaching ATTR-CM is represented by electrocardiogram, which typically shows low voltage in the limb leads or a discrepancy between an augmented left ventricle mass and decreased QRS complex voltages [88]. Moreover, pseudo-infarct Q waves in precordial (especially V1–V3) or inferior leads with normal coronary arteries may be another characteristic sign (Figure 1). Atrial fibrillation and atrioventricular conduction disorders may be common findings in cardiac amyloidosis because of tissue infiltration [89]. 

#### 6.2.2. Echocardiography

**Figure 2:**
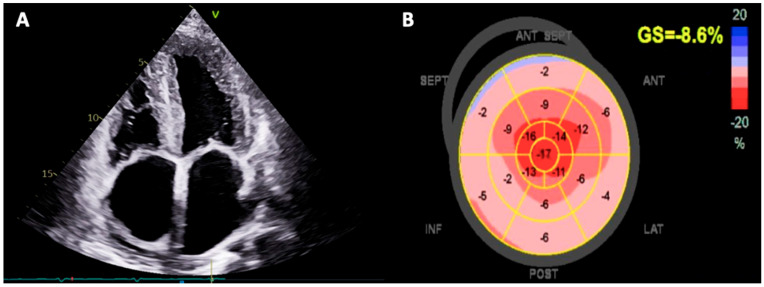
Transthoracic echocardiographic images. Panel (**A**) shows an apical four-chamber view with typical echocardiographic signs of cardiac amyloidosis, i.e., biventricular hypertrophy with granular sparkling pattern, severe bi-atrial enlargement and retro-atrial pericardial effusion. Panel (**B**) shows the global longitudinal strain (GLS) values with the peculiar apical sparing pattern (“cherry on top” pattern), obtained by two-dimensional speckle-tracking echocardiography.

Echocardiography is essential for raising the suspicion of cardiac amyloidosis, as it reveals the presence of left ventricular hypertrophy, defined as an interventricular septum thickness equal to or more than 12 mm, with a granular sparkling appearance (Figure 2A) [90]. Other peculiar echocardiographic red flags are represented by third degree diastolic disfunction with restrictive ventricular filling pattern (E/A > 2 and E/e’ ratio augmented), reduced left ventricular end diastolic volume, reduction of tissue Doppler imaging (TDI) indexes under 5 cm/s, significant bi-atrial enlargement, valve thickening with aortic stenosis, right ventricle hypertrophy and circumferential pericardial effusion [3]. 

In addition, the echocardiographic assessment of global longitudinal strain (GLS) by speckle tracking analysis allows to identify the apical sparing pattern as a distinctive sign of cardiac amyloidosis. Indeed, GLS exhibits a global reduction, usually with the preservation of left ventricular ejection fraction until the latest stages of the disease, with a significant impairment of basal segment contractility and a relative conservation of the apical portions of the left ventricle (Figure 2B) [91]. 

Innovative tools, such as myocardial work (MW) index evaluation, derived from the LV pressure/GLS loop analysis, may play a key role in the future in the differentiation of cardiomyopathies with hypertrophic phenotypes. Among the currently used MW indexes, represented by global work index (GWI), global work efficiency (GWE), global constructive work (GCW) and global wasted work (GWW), it has been demonstrated that GWI and GCW are lower in patients with infiltrative cardiomyopathies in comparison with hypertensive subjects [92]. Moreover, GCW and GWI may represent reliable indexes to distinguish between cardiac amyloidosis and hypertrophic cardiomyopathy, potentially more sensitive than GLS, as they are reduced in patients with ATTR-CM regardless the LV ejection fraction, compared with subjects with hypertrophic cardiomyopathy and with controlled systemic hypertension [93].

In addition, considering that amyloid infiltration affects all the four heart chambers, a specific evaluation of the atria and of the right ventricle with speckle-tracking echocardiography has been performed in patients with CA. It has been demonstrated that left atrial (LA) strain is impaired in subjects with ATTR-CM, and specifically peak left atrial longitudinal strain (LA-PALS) shows an independent association with the diagnosis of cardiac amyloidosis [94,95]. Similarly, right ventricle longitudinal strain and right atrial strain display significantly reduced values in patients with CA, especially ATTR-CM, compared with controls [95,96].

#### 6.2.3. Serum Blood Tests

The alteration of serum blood tests, with an increase in natriuretic peptide levels disproportionate to clinical syndrome and high-sensitivity cardiac troponin chronic elevation without ongoing acute coronary syndrome or any alternative cause, is highly suggestive of amyloidotic cardiomyopathy [97,98]. 

## 7. Diagnosis

**Figure 3:**
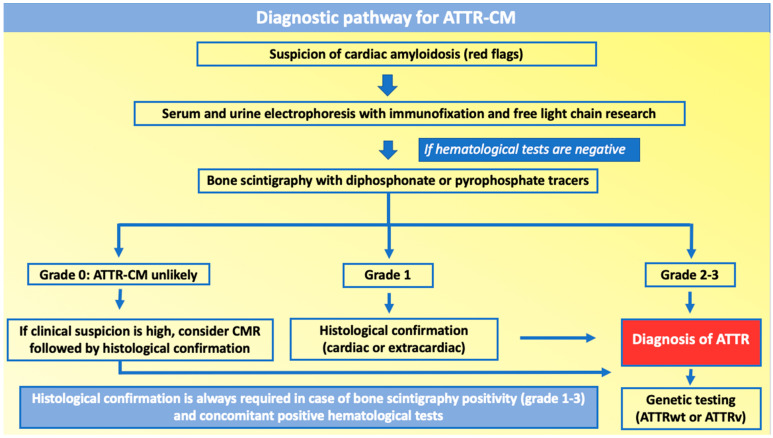
Diagnostic pathway of ATTR-CM. Abbreviations: ATTR-CM—amyloid transthyretin cardiomyopathy; CMR—cardiac magnetic resonance.

The endomyocardial biopsy represents the gold standard diagnostic technique for cardiac amyloidosis [4]. However, a non-invasive diagnostic pathway for the diagnosis of ATTR-CM, not necessarily requiring invasive biopsies, has been established in specific clinical settings, making the diagnosis easier and more accessible, with a relevant impact on the prevalence rates of the disease (Figure 3) [3]. 

### 7.1. Non-Invasive Diagnostic Algorithm

Once the suspicion of cardiac amyloidosis is raised, the first crucial diagnostic step consists in differentiating between ATTR-CM and AL-CM by excluding in ATTR-CM the presence of a monoclonal protein component, through serum and urine protein electrophoresis with immunofixation, along with the research of serum and urine free light chains [3].

The main instrumental diagnostic exam for ATTR-CM detection, both for ATTRwt and ATTRv, is represented by bone scintigraphy with diphosphonate or pyrophosphate tracers, as it shows a peculiar cardiac uptake of the traces, which is linked to the presence of amyloid infiltration in the myocardial extracellular space [99]. 

The significance of myocardial tracer uptake is graduated according to the “Perugini score”. Perugini grade 2 (moderate cardiac uptake greater than bone) and 3 (strong cardiac uptake with little or absent bone signal) are highly specific for ATTR-CM and permit to reach a non-invasive diagnosis of cardiac amyloidosis, whether the monoclonal component is absent and peculiar echocardiographic or cardiac magnetic resonance (CMR) findings are present [3,100]. However, considering that ATTRwt is a senile disease and that benign monoclonal gammopathy prevalence increases in the elderly (approximately 25% of patients), it is not infrequent to have a positive bone scintigraphy with associated positive monoclonal protein. In these cases, before starting a tailored treatment, histological confirmation from cardiac or extracardiac biopsy and amyloid typing are required to determine the actual cause of the cardiomyopathy [101]. 

Perugini grade 1, which stands for mild cardiac uptake without the attenuation of bone uptake, does not allow to reach a definitive diagnosis independently from the results of hematological tests, as it always requires a complementary investigation with a cardiac or extracardiac biopsy (bone marrow, subcutaneous fat, rectum, salivary glands) if the clinical suspect of cardiac amyloidosis is consistent [3]. 

Perugini grade 0, which corresponds to no cardiac uptake of the bone tracer, makes the diagnosis of cardiac amyloidosis very unlikely; nevertheless, some mutations of the ATTR gene, such as Phe64Leu, may determine cardiomyopathy, even if bone scintigraphy does not show any cardiac uptake, requiring definitive histological confirmation if suspicion for ATTR-CM remains high [3,102]. 

In such ambiguous cases, CMR is an important complementary diagnostic tool, which has been included in both invasive and non-invasive diagnostic algorithms [3]. Indeed, the role of CMR has become increasingly important in the field of differential diagnoses of cardiomyopathies with a hypertrophic phenotype, as, on the one hand, it provides reproducible measures of cardiac mass and volumes and of myocardial wall thickness, and, on the other hand, it supplies an accurate characterization of myocardial tissue, resorting to myocardial parametric imaging with T1 and T2 mapping sequences and to the administration of an intravenous contrast medium (gadolinium) [103,104,105].

Specifically, CMR exhibits two specific findings for cardiac amyloidosis, which are deemed essential for the diagnosis, such as a diffuse subendocardial (predominantly) or transmural late gadolinium enhancement, often with atrial involvement, and abnormal gadolinium kinetics with myocardial nulling preceding or coinciding with the blood pool. [3,75]. A diffuse increase in native T1 values, associated with a significant augmentation in extracellular volume fraction (> or equal to 0.40%), are highly evocative but not essential for reaching the diagnosis [3,106].

Regarding a non-invasive diagnosis of CA, innovative diagnostic techniques have emerged in the last years, such as PET/CT scan with [18F]-Florbetaben, which has displayed promising premises for the non-invasive differential diagnosis between AL-CM and ATTR-CM [107]. The analysis of PET/CT scans of patients with AL-CM, ATTR-CM and controls without CA, demonstrated that, after an intense early cardiac uptake in all the patients, an elevated and persistent cardiac uptake was present in subjects with AL-CM, in contrast with the rapid uptake decrease after the precocious scan observed in subjects affected by ATTR-CM and patients without CA [107].

### 7.2. Invasive Diagnostic Tools

Whether the non-invasive diagnostic algorithm fails to reach a certain diagnosis, either because of the coexistent positivity of hematological tests and bone scintigraphy or in case of inconclusive bone scintigraphy (Perugini grade 0–1) but with consistent clinical and radiological (CMR) suspect of CA, histological confirmation by a cardiac or extra-cardiac biopsy is needed [3]. 

The gold standard invasive diagnostic technique is represented by endomyocardial biopsy with Congo red staining, which, however, exhibits several limitations. It is a risky and expensive procedure that may not always be promptly available, determining consistent diagnostic delay, as it should be conducted in highly specialized centers [108]. Beyond logistical limitations, given that cardiac amyloidosis may determine a regional patchy myocardial involvement, endomyocardial biopsy shows intrinsic pitfalls linked to undersampling, as it is usually restricted to the right atrium and to the right ventricle portion of the interventricular septum [109,110].

In consideration of the above-mentioned limitations, an extracardiac biopsy may represent a valid alternative to reach the diagnosis. Indeed, according to the current diagnostic algorithm, the positivity of an extracardiac biopsy, associated with echocardiographic or CMR typical features, allows to formulate the diagnosis of cardiac amyloidosis [3]. Several tissues, including subcutaneous fat, rectum and salivary glands, may be targeted [1]. Specifically, abdominal fat pad fine needle aspiration (FPFNA) allows to overcome the risks of major complications, the elevated costs and the scarce availability associated with endomyocardial biopsy, as it is an easy, secure and cheap procedure that may be performed bedside [111]. 

FPFNA is a widely used well-established technique in the setting of systemic amyloidosis. Indeed, it shows a high sensibility in AL-CM with elevated rates of positivity to Congo Red staining of specimens, directly proportional to the systemic amyloid burden (up to 100% in patients with large and diffuse amyloid infiltration), obviating the need to resort to endomyocardial biopsy [108]. 

In contrast, the diagnostic role of FPFNA in the field of ATTR-CM is still debated as it exhibits a lower sensibility for ATTR-CM detection, corresponding to 15% of patients with ATTRwt and to 45% of subjects affected by ATTRv, with different rates depending on genetic mutation and the extent of extracardiac involvement [108,112]. Indeed, similarly to AL-CM, a higher systemic amyloid burden in ATTRv, given by the involvement of peripheral and autonomic nerves, determines higher rates of positivity for amyloid of FPFNA samples [108]. 

### 7.3. Genetic Testing

Once a diagnosis of ATTR-CM is confirmed, genetic testing is always required to distinguish between ATTRwt and ATTRv, although clinical and epidemiological evidence may give some clues, as ATTRv may be associated with peripheral polyneuropathy and affects younger patients more than ATTRwt, which more often occurs in elderly male patients [113].

## 8. Treatment

### 8.1. Specific Treatment

Based on the extensive studies regarding TTR pathophysiology, currently, two main tailored therapeutic approaches are available for clinical use in ATTR-CM.

The first strategy is founded on the idea of stabilizing the tetrameric structure of TTR, as tetramer dissociation into amyloidogenic fragments represents the first pathogenetic moment that initiates the cascade of events leading to insoluble amyloid fibrils formation [114]. Diflunisal has been the first tetramer stabilizer. Tafamidis and acoramidis belong to this category and represent the leading therapeutic strategies in ATTRwt cardiomyopathy, as clinical trials have shown significant benefit regarding all-cause death and hospitalization for cardiac issues [115,116]. 

The second therapeutic approach is constituted by TTR gene silencer agents, which aim to reduce the amyloid burden by limiting the production of TTR, predominantly at the hepatic level [3]. Two principal mechanisms of action are exploited, represented by small interfering RNAs (such as patisiran and vutrisiran) and anti-sense oligonucleotide (inotersen) [113]. As randomized clinical trials have shown an improvement in neurologic symptoms and quality of life, these drugs are currently used in ATTRv with overt neuropathy [117,118,119]. However, recently, the APOLLO B trial has demonstrated that the use of patisiran in patients with ATTR cardiomyopathy has beneficial effects on functional capacity, limiting the decline in the 6-min walk distance and improving general health status, measured by the Kansas City Cardiomyopathy Questionnaire Overall Summary [120]. 

Notably, a third therapeutic strategy based on the clearance of amyloid deposits through specific selective anti-TTR monoclonal antibodies may soon be available, as clinical trials are ongoing and show promising results from preliminary data [3]. The antibodies (PRX004, NI006) bind selectively pathological amyloid fibrils and stimulate the activation of the macrophage to facilitate the elimination of amyloid deposits. In phase 1 trials, NI006 reduced the amyloid burden, measured through the reduction of both tracer uptake at the cardiac level on scintigraphy and levels of ECV on CMR, with no significant adverse effects [121]. 

Other specific gene-editing therapeutic agents, such as NTLA-2001, have been tested on six patients with ATTRv. This approach aims to decrease serum TTR protein levels with a targeted knockout of transthyretin, through the clustered regularly interspaced short palindromic repeats and associated Cas9 endonuclease (CRISPR-Cas9) system. Preliminary results have been encouraging, with mild adverse effects and a reduction in TTR plasmatic concentration [122].

### 8.2. Treatment of Comorbidities 

Management of HFpEF should follow the current guidelines, avoiding, however, the use of angiotensin converting enzyme (ACE) inhibitors and angiotensin receptor blockers, because of the tendency of hypotension seen in these patients [3]. Particular attention should be paid to the dosage of beta-blockers, which should be managed with caution, as these drugs could exacerbate the atrioventricular conduction abnormalities linked to the amyloid infiltration of the electrical conduction system, facilitating the appearance of high-degree atrioventricular blocks, requiring pacemaker implantation [1].

Atrial fibrillation represents a common finding in ATTR-CM and exhibits a high rate of recurrence after electrical cardioversion or ablation. Regarding medical therapy, amiodarone is the preferred anti-arrhythmic drug and anticoagulation is recommended regardless the CHA2DS2-VASc score [5]. 

Aortic stenosis is a frequent finding in CA, associated with worse clinical outcomes. Its therapeutic management consists of transcatheter valve replacement, which has demonstrated to improve prognosis [56].

## 9. Conclusions

The rise in clinical awareness of transthyretin cardiac amyloidosis, along with the widespread use of sensitive imaging techniques, has led to an increase in the amount of diagnosis during the last years.

Nevertheless, the real prevalence of the disease may still be significantly underestimated, as suggested by the autoptic case series, which unveiled the presence of several cases of amyloid deposition in cardiac tissues in elderly patients, with a ratio of up to 29%.

Integrating evidence from clinical data and forensic studies, it is plausible that, still today, many cases of cardiac ATTR remain misdiagnosed, and, thus, it is crucial to increase attention towards the clinical red flags to make early diagnoses and initiate the available tailored disease-modifying treatments.

## Figures and Tables

**Table 1:** Main epidemiological data from registries of diagnosed patients. N/A—not applicable.

Authors	Year of Publication	Setting	Geographic Area	Date of Study Beginning	Prevalence Rate at Beginning	Date of Study Ending	Prevalence Rate at End	Sex Distribution
Lauppe et al. [53]	2021	National registries	Sweden	2008	1.0 per 100,000 person-years	2018	5.0 per 100,000 person-years	30% female
Lauppe et al. [54]	2022	National registries	Norway	2018	1.1 per 100,000 person-years	2018	3.7 per 100,000 person-years	24.4% female
Cappelli et al. [55]	2023	Regional registries	Tuscany	N/A	N/A	2022	90.3 per 1,000,000 persons for ATTRwt 9.5 per 1,000,000 persons for ATTRv	Predominance of male sex
Gilstrap et al. [56]	2019	Medicare beneficiaries registries	United States of America	2000	8 to 17 per 100,000 person-years	2012	18 to 55 per 100,000 person-years	51.7% female

**Table 2:** Main data regarding registry epidemiological data from autoptic studies. N/A*—not applicable. HFpEF—heart failure with preserved ejection fraction.

Authors	Year of Publication	Methods of the Study	Subjects	Cardiac Amyloidosis Prevalence	ATTR-CM (% of CA Patients)	Sex Distribution
Lie et al. [72]	1988	Autopsy on unselected deceased humans 90–105 years old	237	21%	100	61% female
Roberts et al. [73]	1998	Autopsy on unselected deceased humans ≥ 80 years old	490	4%	N/A*	49% female
Tanskanen et al. [74]	2008	Autopsy on unselected deceased humans > 85 years old	256	5%	100	N/A*
Porcari et al. [75]	2021	Autopsy on unselected deceased humans ≥ 75 years old	56	29%	50	57% female
Corwell et al. [76]	1983	Autopsy on unselected deceased humans ≥ 80 years old	85	25%	100	47% female
Mohammed et al. [77]	2014	Autopsy on patients with antemortem-diagnosed HFpEF	109	5% (moderate to severe amyloid deposition)	N/A*	80% males
